# Ceragenins and Antimicrobial Peptides Kill Bacteria through Distinct Mechanisms

**DOI:** 10.1128/mbio.02726-21

**Published:** 2022-01-25

**Authors:** Gabriel Mitchell, Melanie R. Silvis, Kelsey C. Talkington, Jonathan M. Budzik, Claire E. Dodd, Justin M. Paluba, Erika A. Oki, Kristine L. Trotta, Daniel J. Licht, David Jimenez-Morales, Seemay Chou, Paul B. Savage, Carol A. Gross, Michael A. Marletta, Jeffery S. Cox

**Affiliations:** a Department of Molecular and Cell Biology, University of California, Berkeley, California, USA; b Department of Microbiology and Immunology, University of California, San Francisco, California, USA; c Department of Medicine, University of California, San Francisco, California, USA; d Department of Biochemistry and Biophysics, University of California, San Francisco, California, USA; e Department of Medicine, Division of Cardiovascular Medicine, Stanford University, California, USA; f Department of Cellular and Molecular Pharmacology, University of California, San Francisco, California, USA; g Department of Chemistry and Biochemistry, Brigham Young University, Provo, Utah, USA; h Department of Cell and Tissue Biology, University of California, San Francisco, California, USA; i Department of Chemistry, University of California, Berkeley, California, USA; j California Institute for Quantitative Biosciences, University of California, Berkeley, California, USA; k Chan Zuckerberg Biohub, San Francisco, California, USA; McMaster University

**Keywords:** Antibiotics, Gram-negative, Gram-positive, mycobacteria, CSA

## Abstract

Ceragenins are a family of synthetic amphipathic molecules designed to mimic the properties of naturally occurring cationic antimicrobial peptides (CAMPs). Although ceragenins have potent antimicrobial activity, whether their mode of action is similar to that of CAMPs has remained elusive. Here, we reported the results of a comparative study of the bacterial responses to two well-studied CAMPs, LL37 and colistin, and two ceragenins with related structures, CSA13 and CSA131. Using transcriptomic and proteomic analyses, we found that Escherichia coli responded similarly to both CAMPs and ceragenins by inducing a Cpx envelope stress response. However, whereas E. coli exposed to CAMPs increased expression of genes involved in colanic acid biosynthesis, bacteria exposed to ceragenins specifically modulated functions related to phosphate transport, indicating distinct mechanisms of action between these two classes of molecules. Although traditional genetic approaches failed to identify genes that confer high-level resistance to ceragenins, using a Clustered Regularly Interspaced Short Palindromic Repeats interference (CRISPRi) approach we identified E. coli essential genes that when knocked down modify sensitivity to these molecules. Comparison of the essential gene-antibiotic interactions for each of the CAMPs and ceragenins identified both overlapping and distinct dependencies for their antimicrobial activities. Overall, this study indicated that, while some bacterial responses to ceragenins overlap those induced by naturally occurring CAMPs, these synthetic molecules target the bacterial envelope using a distinctive mode of action.

## INTRODUCTION

Our current arsenal of antibiotics will soon be ineffective against the simplest bacterial infections due to the continued spread of antibiotic resistance (AR) (1). AR has been identified in virtually all bacterial species of clinical relevance, including Gram-positive and Gram-negative bacteria as well as mycobacteria (2). Despite the threat that AR represents to global health, there is a lack in the development of antimicrobials with innovative mechanisms of action (3–5). A better understanding of the fundamental principles of how antibiotics kill microbes and how AR develops is key to breaking the futile cycle of antibiotic development and microbial evolution.

Antimicrobial peptides are structurally diverse molecules expressed in a wide array of organisms that directly kill microbes, including bacteria (6, 7). Many antimicrobial peptides, such as the class of cationic antimicrobial peptides (CAMP), rapidly kill bacteria likely by disrupting membranes, although other mechanisms of action have been suggested (7–9). The potential of using CAMPs to treat AR infections has become a research focus due to their action against a broad spectrum of pathogens, their selectivity toward microbial membranes, and the slow development of resistance (6, 10). Despite some progress in this area, significant barriers to CAMP therapeutic development include high production costs, toxicity, susceptibility to proteolytic degradation, and activation of allergic responses (6, 10).

Ceragenins are a family of synthetic amphipathic molecules derived from cholic acid designed to mimic the activity of endogenous CAMPs (11, 12). These molecules are inexpensive to manufacture and are not susceptible to proteolysis, making them an attractive alternative to peptide-based synthetic CAMPs (11). Importantly, ceragenins have antimicrobial activity against a broad spectrum of microbes, which include both Gram-negative and Gram-positive bacteria (11). High-level resistance to ceragenins is seemingly difficult to acquire in the lab as attempts to isolate ceragenin-resistance bacterial mutants failed in the Gram-positive bacterium Staphylococcus aureus and identified only modest and unstable resistance in Gram-negative organisms (13). Although ceragenins were designed as CAMP mimics and can depolarize bacterial membranes (14), the inability to identify bonafide ceragenin-resistant bacterial mutants represents a major barrier in understanding their mechanism of action.

Here, we took a comparative strategy and used a combination of transcriptomic, proteomic, and genetic approaches to study the bacterial responses to treatment with ceragenins and two well-studied CAMPs. The results of this study suggested that ceragenins kill bacteria by disrupting the bacterial envelope through a distinctive mode of action from naturally occurring CAMPs. We also showed that ceragenins have activity against mycobacteria despite their unique cell wall architecture.

## RESULTS

### Susceptibility of bacteria to CAMPs and ceragenins.

**FIG 1:**
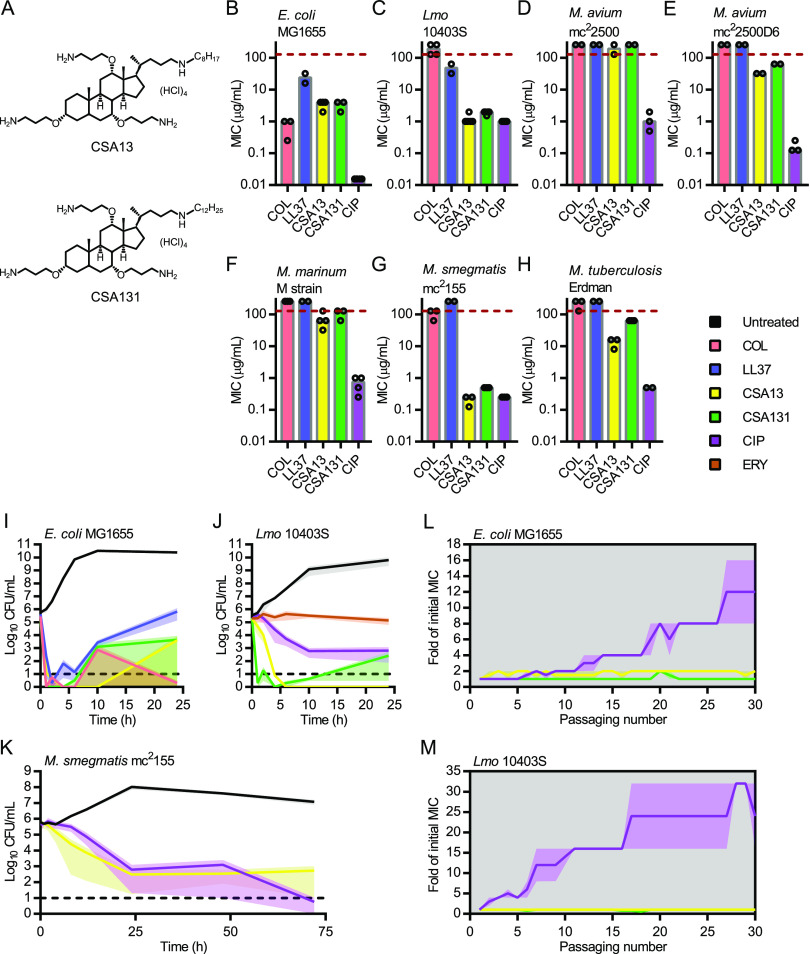
Ceragenins kill phylogenetically diverse bacteria. (A) Structures of the ceragenins CSA13 and CSA131. MICs of colistin (COL), LL37, CSA13, CSA131, and ciprofloxacin (CIP) against E. coli MG1655 (B), L. monocytogenes (Lmo) 10403S (C), M. avium mc^2^2500 (D), M. avium mc^2^2500D6 (E), M. marinum M strain (F), M. smegmatis mc^2^155 (G) and M. tuberculosis Erdman (H). Dots and bars indicate results from independent experiments and median values, respectively. Time-kill experiments of E. coli MG1655 (I), L. monocytogenes 10403S (J), and M. smegmatis mc^2^155 (K) exposed to colistin (in pink), LL37 (in blue), CSA13 (in yellow), CSA131 (in green), ciprofloxacin (in purple) and/or erythromycin (ERY; in orange), a bacteriostatic antibiotic. Untreated samples are in black, and results are shown as means from two independent experiments. Shaded areas show standard error of the mean (SEM), and dotted lines indicate the limit of detection. Serial passages of E. coli (L) and L. monocytogenes (M) exposed to CSA13 (in yellow), CSA131 (in green), and ciprofloxacin (in purple). Bacteria were passaged daily in the presence of subinhibitory concentrations of antibiotics. Results are expressed as means and SEM from two independent experiments.

MICs for the CAMPs colistin and LL37 as well as two ceragenin compounds, CSA13 and CSA131 (see structures in Fig. 1A), were determined against the Gram-negative bacterium E. coli, the Gram-positive bacterium Listeria monocytogenes and several mycobacterial species (i.e., Mycobacterium avium, M. marinum, M. smegmatis, and M. tuberculosis) (Fig. 1B to H and Table S1). M. avium mc^2^2500 and mc^2^2500D are two colony morphotypes of a clinical strain that predominantly form either smooth/transparent (mc^2^2500) or opaque (mc^2^2500D) colonies (15). The fluoroquinolone antibiotic ciprofloxacin (CIP), which inhibits DNA gyrase, was included as a positive-control. As expected, colistin, which requires binding to lipopolysaccharide (LPS) for activity (16), was active against E. coli (Fig. 1B) but not against L. monocytogenes (Fig. 1C) or any of the mycobacterial species (Fig. 1D to H). Interestingly, LL37 was active against E. coli (Fig. 1B) and L. monocytogenes (Fig. 1C) but had no detectable activity against mycobacterial species (Fig. 1D to H). The ceragenins CSA13 and CSA131 were also active against both E. coli (Fig. 1B) and L. monocytogenes (Fig. 1C). In contrast to colistin and LL37, the ceragenins had activity against mycobacteria, although the MICs varied between species (Fig. 1D to H). While M. smegmatis was highly susceptible to CSA13 and CSA131 (Fig. 1G), both compounds were less active against the slower-growing species M. avium (Fig. 1D and E), M. marinum (Fig. 1F), and M. tuberculosis (Fig. 1H). Similar trends in MIC values for E. coli, L. monocytogenes, and M. smegmatis were observed with two other structurally related ceragenin compounds, CSA44 and CSA144 (See Table S1), which further confirmed that ceragenins have antimicrobial activity against mycobacteria. Overall, these results demonstrate that the spectrum of activity of ceragenins is broader than colistin and LL37, indicating different requirements for activity.

10.1128/mBio.02726-21.1 TABLE S1 Minimal inhibitory concentrations (MIC) of colistin, LL37, CSA13, CSA131, CSA44, CSA144, and ciprofloxacin against E. coli, L. monocytogenes, and Mycobacterium spp. Download Table S1, PDF file, 0.01 MB. Copyright © 2022 Mitchell et al. 2022 Mitchell et al. https://creativecommons.org/licenses/by/4.0/ This content is distributed under the terms of the Creative Commons Attribution 4.0 International license.

### Ceragenins are bactericidal.

To determine if ceragenins kill all three types of bacteria, we performed survival experiments at inhibitory concentrations (∼1 to 2 × MICs) of the molecules (Fig. 1I to K). Like colistin, LL37 and ciprofloxacin, ceragenin treatment (CSA13 or CSA131) led to cell killing of E. coli (Fig. 1I), L. monocytogenes (Fig. 1J), and M. smegmatis (Fig. 1K), although some bacteria began to grow slightly by the end of the culturing, perhaps due to inactivation of the antibiotic or the emergence of resistant bacteria at these later time points. This late regrowth phenotype seemed to be highly dependent on antibiotic concentration and was not observed in a L. monocytogenes culture treated with a 2-fold higher concentration (4 μg/mL) of CSA131 (data not shown). The bactericidal activities of CAMPs, ceragenins, and ciprofloxacin are in contrast with the activity of the bacteriostatic antibiotic erythromycin against L. monocytogenes (Fig. 1J), which did not cause any significant reduction in cell number but inhibited bacterial growth. Overall, these results confirmed that ceragenins act on bacteria through a bactericidal mechanism.

### Serial passage of E. coli and L. monocytogenes in the presence of subinhibitory concentrations of ceragenins.

Isolation and characterization of antibiotic-resistant bacteria could provide insight into the mode of action of ceragenins. Although a previous study showed that bacterial resistance to ceragenins is infrequently observed *in vitro* and unstable (13), the late regrowth of bacteria that was observed when they were exposed to ceragenins during the previous survival experiments (Fig. 1I and J) led us to attempt generating ceragenin-resistant bacteria. To this end, we performed serial passaging experiments with E. coli and L. monocytogenes in the presence of ciprofloxacin, CSA13, and CSA131 (Fig. 1L and M). During these experiments, bacteria were cultured overnight in the presence of a range of antimicrobial concentrations, and bacteria that grew at the highest concentration of antimicrobials (right below MIC) were used as inoculums for the next growth cycle. A total of 30 passages were performed for both bacterial species, and antibiotic susceptibilities were determined and recorded for each of these passages to monitor the gradual emergence of resistance to the antimicrobials. In contrast to ciprofloxacin-exposed bacteria, E. coli and L. monocytogenes bacteria exposed to ceragenins did not give rise to stable resistance (Fig. 1L and M). The generation of spontaneous M. smegmatis mutants resistant to CSA13 was also attempted, but no CSA13-resistant bacteria were recovered, although bacteria resistant to ciprofloxacin and rifampicin were isolated from parallel control experiments (data not shown). These results confirmed that resistance to ceragenins is infrequent (13) and does not emerge *in vitro* under conditions known to generate resistant mutants against other antibiotics.

### Transcriptional response of E. coli exposed to ceragenins.

**FIG 2:**
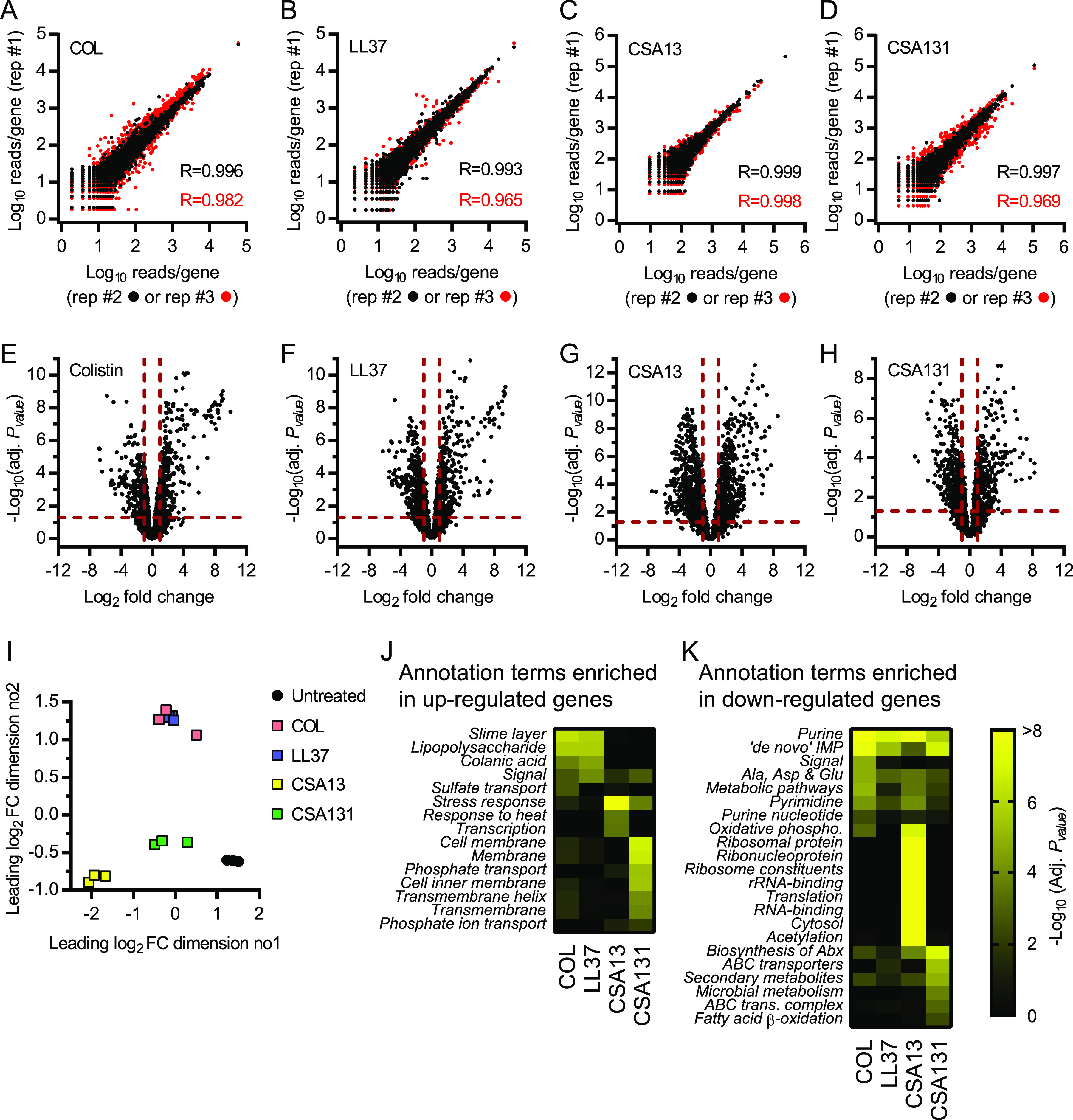
Transcriptional response of E. coli exposed to ceragenins. RNA from exponentially growing E. coli bacteria exposed to supra-MICs of colistin (COL), LL37, CSA13, and CSA131 was extracted and sequenced. (A–D) Replica plots showing the log_10_ of the normalized number of reads per gene for bacteria exposed to antibiotics. Correlation coefficients (R) between replicates #1 and #2 (black dot) and #1 and #3 (red dot) are displayed. (E-H) Volcano plots that represent RNA expression as means of log_2_-fold change and -log_10_ adjusted *P* values (adj. *P* value) for bacteria exposed to antibiotics in comparison to untreated control samples. Horizontal and vertical dotted red lines indicate adjusted *P* values less than 0.05 (or −log_10_ [adj. *P* value] greater than 1.3) and absolute log_2_-fold change greater than 1. (I) Multidimensional scaling (MDS) plot showing the separation between biological replicates and between untreated and antibiotic-treated samples. (J) Annotation terms enriched for genes significantly upregulated (log_2_ FC > 1 and adj. *P* value <0.05) following exposure to antibiotics. (K) Annotation terms enriched for genes significantly downregulated (log_2_ FC <−1 and adj. *P* value <0.05) following exposure to antibiotics. Adjusted *P* values of annotation terms associated with a false discovery rate (FDR) value >0.05 for at least one antibiotic are shown. Only the 8 most statistically significant annotation terms are shown for each condition. Annotation terms are abbreviated and/or modified for a purpose of presentation (see Data Set S2 for original annotation terms). Data are from 3 independent experiments.

To gain insights into the mechanism of action of CAMPs and ceragenins, we determined the global transcriptional responses of E. coli exposed to colistin, LL37, CSA13, and CSA131 using RNAseq, as similarly used for other antibacterial compounds (17, 18). Bacteria were grown to log phase and then treated with supra-MICs of antibiotics for 1 h before RNA extraction and sequencing (see Materials and Methods). Plots of the normalized number of reads per gene showed excellent correlation (*R* = 0.965 to 0.999) between biological replicates for each of the conditions tested (Fig. 2A to D), demonstrating the reproducibility of the method. Hundreds of statistically significant changes in gene expression (defined by absolute log_2_-fold change >1 and adjusted *P* value <0.05) following exposure of bacteria to colistin, LL37, CSA13, and CSA131 were measured (Fig. 2E to H and Data Set S1).

10.1128/mBio.02726-21.4 DATA SET S1 Transcriptional responses of E. coli exposed to colistin, LL37, CSA13, and CSA131 as determined by RNAseq. This file includes raw and normalized read counts, fold changes, *P* values, adjusted *P* values, and lists of genes significantly up- or down-regulated. Download Data Set S1, XLSX file, 1.7 MB. Copyright © 2022 Mitchell et al. 2022 Mitchell et al. https://creativecommons.org/licenses/by/4.0/ This content is distributed under the terms of the Creative Commons Attribution 4.0 International license.

10.1128/mBio.02726-21.5 DATA SET S2 Annotation term enrichment analysis for significantly up- or down-regulated genes in E. coli exposed to colistin, LL37, CSA13, and CSA131. The analysis was performed with the DAVID Bioinformatics Resources using terms from UniProt keywords, COG ontology, GO, and the KEGG pathway. Download Data Set S2, XLSX file, 0.5 MB. Copyright © 2022 Mitchell et al. 2022 Mitchell et al. https://creativecommons.org/licenses/by/4.0/ This content is distributed under the terms of the Creative Commons Attribution 4.0 International license.

The global transcriptional responses of E. coli to CAMPs and ceragenins were analyzed using multidimensional scaling analysis. Interestingly, while transcriptional responses of bacteria to the CAMPs colistin and LL37 were essentially the same, the response to CSA13 and CSA131 were not only distinct from these CAMPs but also distinct from each other (Fig. 2I). Pathway analysis corroborated that E. coli responded differently to CAMPs and ceragenins and showed enrichment of annotation terms associated with the outer membrane (e.g., lipopolysaccharide and colanic acid) in genes upregulated by CAMPs but not ceragenins (Fig. 2J and Data set S2). Pathway analysis also corroborated the heterogeneity in the bacterial response to ceragenins and showed enrichment of terms associated with translation in genes downregulated by CSA13 but not CSA131 (Fig. 2K and Data Set S2). While the two ceragenins are structurally quite similar, the addition of four methylene groups to the CSA13 carbon chain to create CSA131 significantly alters the hydrophobicity of the molecule, increasing the partition coefficient from log P_CSA13_ = 5.51 to log P_CSA131_ = 7.29 (see Materials and Methods), which likely contributes to differences in antibacterial activities and bacterial responses. Overall, these results showed that transcriptional responses of E. coli to the naturally occurring CAMPs colistin and LL37 are similar but differ from the response to ceragenins, identifying for the first time that these CAMP mimics have a distinct function. These results also suggested that E. coli responds differently to the structurally related ceragenin compounds, CSA13 and CSA131.

### Identification of pathways defining the transcriptional response of E. coli to ceragenins.

**FIG 3:**
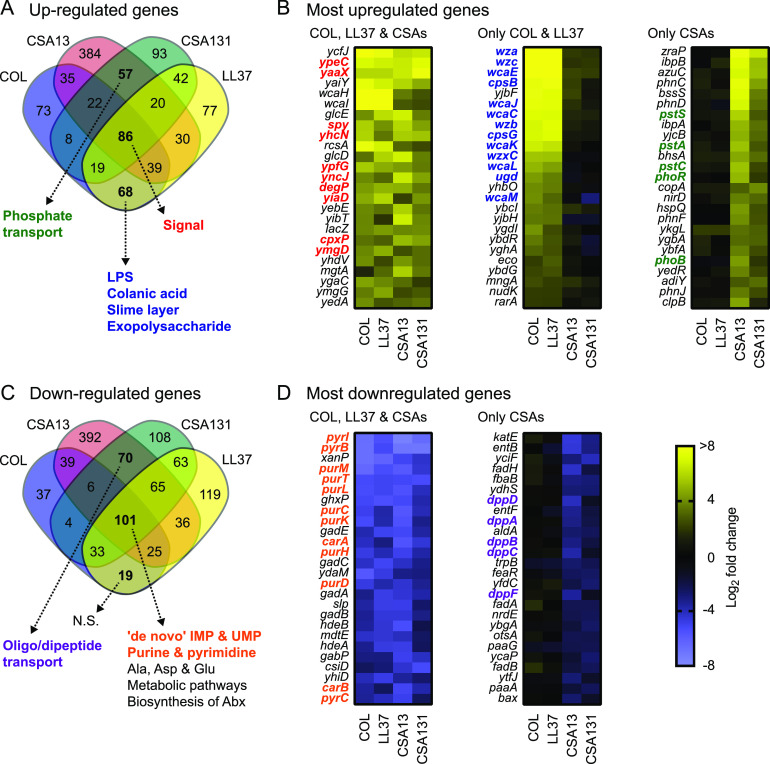
Identification of pathways defining the transcriptional response of E. coli to ceragenins. (A) Venn diagram analysis of genes significantly upregulated (log_2_ FC >1 and adjusted *P* value <0.05) in E. coli exposed to antibiotics. Annotation terms associated with a false discovery rate (FDR) value > 0.05 for genes upregulated by all antibiotics, by CAMPs, or by ceragenins are shown by dotted arrows. (B) Top 25 most upregulated genes for bacteria exposed to all antibiotics (COL, LL37 & CSAs), CAMPs (COL & CSAs), or ceragenins. Genes belonging to the annotation terms signal (in red), LPS, colanic acid, slime layer, and exopolysaccharide (in blue), and phosphate transport (in green) are indicated. (C) Venn diagram analysis of genes significantly downregulated (log_2_ FC <−1 and adjusted *P* value <0.05) in E. coli exposed to antibiotics. Annotation terms associated with a false discovery rate (FDR) value > 0.05 for genes downregulated by all antibiotics, by CAMPs, or by ceragenins are shown by dotted arrows. (D) Top 25 most downregulated genes for bacteria exposed to all antibiotics or ceragenins. Genes belonging to the annotation terms “*de novo* IMP”, “*de novo* UMP”, “purine”, and “pyrimidine” (in orange), and “oligonucleotide/dipeptide transport” (in purple) are indicated. Annotation terms are abbreviated and/or modified for a purpose of presentation (see Data Set S3 for original annotation terms). Data are from 3 independent experiments.

Pathway analysis of genes modulated by more than one compound was performed to further define transcriptional responses to CAMPs and ceragenins. The Venn diagram in Fig. 3A visualizes the extent of overlap of the individual E. coli genes that had significant increases in mRNA abundance upon treatment with each of the molecules. In particular, 86 genes were induced in all 4 conditions, 68 were upregulated specifically during CAMP treatment while 57 were induced by the ceragenins (Fig. 3A and Data Set S3). The annotation term “signal” was significantly enriched among the genes upregulated by all antibiotics (Fig. 3A and Data Set S3), which is indicative of a common response to CAMPs and ceragenins. Interestingly, this group included genes involved in the membrane stress response such as *spy*, *degP*, and *cpxP* (19) (Fig. 3B). Consistent with results from Fig. 2, genes specifically upregulated in bacteria exposed to CAMPs were significantly associated with annotation terms related to LPS/colanic acid biosynthesis and included genes such as *wzc*, *wcaE*, and *cpsB* (Fig. 3A and B and Data Set S3). Interestingly, genes specifically upregulated in bacteria exposed to ceragenins were associated with phosphate transport (Fig. 2J, Fig. 3A, and Data Set S3) and included genes encoding the major phosphate-responsive regulators PhoR and PhoB (Fig. 3B). Overall, these results suggested that E. coli responds to CAMPs and ceragenins by upregulating genes involved in signaling and the response to membrane stress. These results also showed that, while exposure of E. coli to CAMPs specifically induces the expression of genes related to LPS and colanic acid biosynthesis, exposure to ceragenins uniquely induces the expression of genes involved in phosphate transport.

10.1128/mBio.02726-21.6 DATA SET S3 Venn diagram lists and annotation term enrichment analysis for genes significantly up- or down-regulated in E. coli by all antibiotics, by CAMPs, or by ceragenins. The analysis was performed with the DAVID Bioinformatics Resources using terms from UniProt keywords, COG ontology, GO, and the KEGG pathway. Download Data Set S3, XLSX file, 0.10 MB. Copyright © 2022 Mitchell et al. 2022 Mitchell et al. https://creativecommons.org/licenses/by/4.0/ This content is distributed under the terms of the Creative Commons Attribution 4.0 International license.

For genes with mRNA levels that decreased during these treatments, 101 were downregulated by all antibiotics, 19 by CAMPs, and 70 by ceragenins (Fig. 3C and Data Set S3). Pathway analysis identified significant enrichment of terms related to amino acids and nucleotide metabolism in genes downregulated following exposure to all antibiotics (Fig. 3C and Data Set S3). This was corroborated by the finding that genes related to purine and pyrimidine biosynthesis (e.g., *pyrB*, *purM*, and *purT*) were among the most significantly downregulated genes by these antimicrobials (Fig. 3D), which is consistent with our pathway analysis (Fig. 2K). Genes downregulated by ceragenins showed enrichment for genes involved in oligopeptide/dipeptide transport such as *dppD* and *dppA* (Fig. 3C, Fig. 3D, and Data Set S3). Although the reason for the downregulation of genes involved in peptide transport in E. coli exposed to ceragenins is unknown, these results suggested that bacteria exposed to CAMPs and ceragenins respond by downregulating genes involved in metabolic pathways.

### Identification of cis-acting elements that regulate the transcriptional response of E. coli to ceragenins.

**FIG 4:**
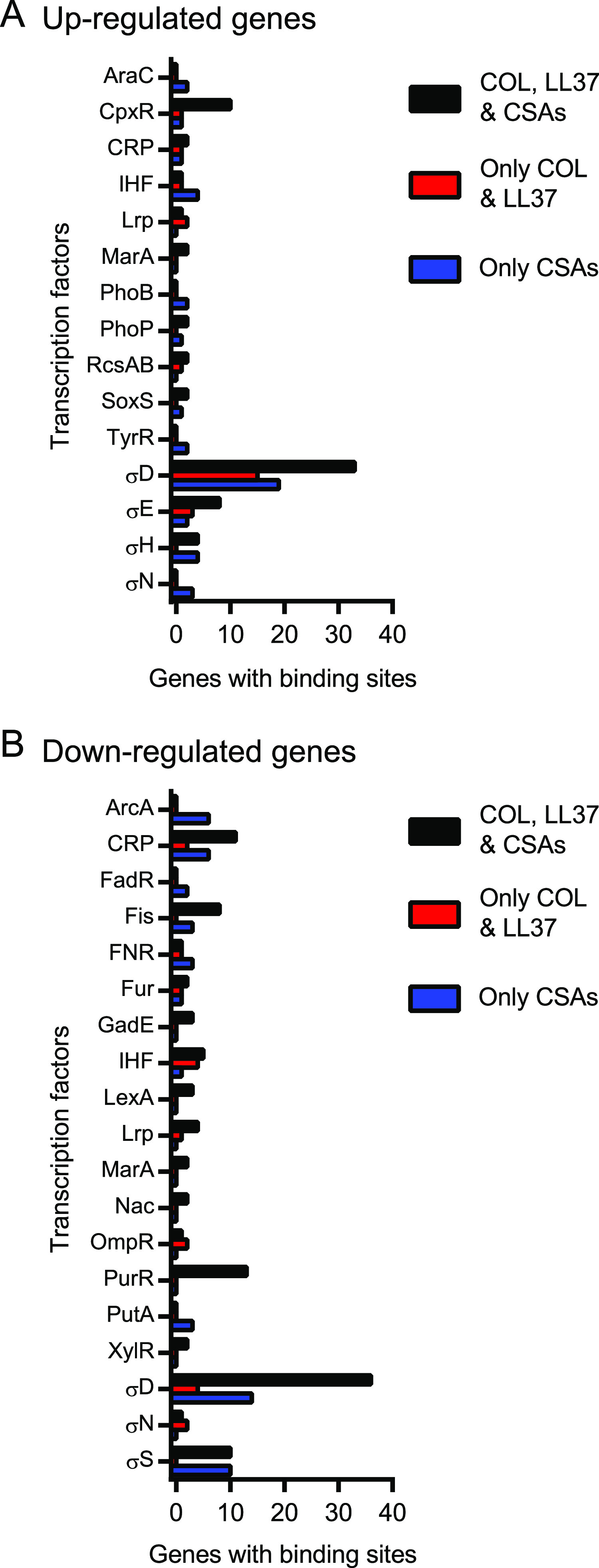
Identification of cis-acting elements that regulate the transcriptional response of E. coli to ceragenins. Genes with known or predicted promoters or binding sites for transcription factors are enumerated for genes commonly upregulated (A) or downregulated (B) by all antibiotics (black bars), by CAMPs (red bars), or by ceragenins (in blue). Only promoters and binding sites identified more than once for at least one group are represented.

To determine which signal transduction pathways control the transcriptional responses to CAMPs and ceragenins, the DNA sequences immediately 5′ of genes with significantly altered mRNA levels were analyzed for the presence of cis-acting promoter and operator sequences known or predicted to bind transcriptional regulators (20). Interestingly, genes upregulated following exposure to each of the molecules (Fig. 4A and Data Set S4) were associated with cis-acting elements that interact with the response regulator CpxR of the CpxA/CpxR two-component regulatory system (enrichment of 11.63%; 10 out of 86 genes), which responds to the envelope stress (21) and is consistent with the upregulation of the CpxR regulon genes *spy*, *degP* and *cpxP* (Fig. 3B). This analysis also showed enrichment for genes associated with cis-acting elements binding the primary sigma factor σ^D^ (22), involved in the redistribution of the RNA polymerase in response to osmotic stress (23), the alternative sigma factor σ^E^ that coordinates the envelope stress response (21, 24, 25), and the alternative sigma factor σ^H^, which controls the expression of heat shock genes and genes involved in membrane functionality and homeostasis (26) (Fig. 4A). Genes downregulated following exposure to all antibiotics were associated with the presence of binding sites for the HTH-type transcriptional repressor PurR (Fig. 4B and Data Set S4), which regulates genes involved in the *de novo* synthesis of purine and pyrimidine nucleotides (27, 28), and corroborated our analysis above (Fig. 2K, and 3C and D). Overall, these results suggested that CAMPs and ceragenins perturb the bacterial envelope and trigger the CpxA/CpxR system. These results also suggested that PurR downregulates the expression of genes involved in the biosynthesis of purine and pyrimidine following the exposure of E. coli to CAMPs and ceragenins.

10.1128/mBio.02726-21.7 DATA SET S4 Analysis of cis-acting elements associated with genes significantly up- or down-regulated in E. coli exposed to colistin, LL37, CSA13, and CSA131. This file includes lists of genes with a least one binding site for sigma factors or transcription factors as well as numbers of nonredundant binding sites for each condition. Fold changes for genes of the CpxR, PurR, RcsA, and PhoB regulons are also included. Download Data Set S4, XLSX file, 0.04 MB. Copyright © 2022 Mitchell et al. 2022 Mitchell et al. https://creativecommons.org/licenses/by/4.0/ This content is distributed under the terms of the Creative Commons Attribution 4.0 International license.

### Expression of the CpxR, PurR, RcsA, and PhoB regulons in E. coli exposed to ceragenins.

**FIG 5:**
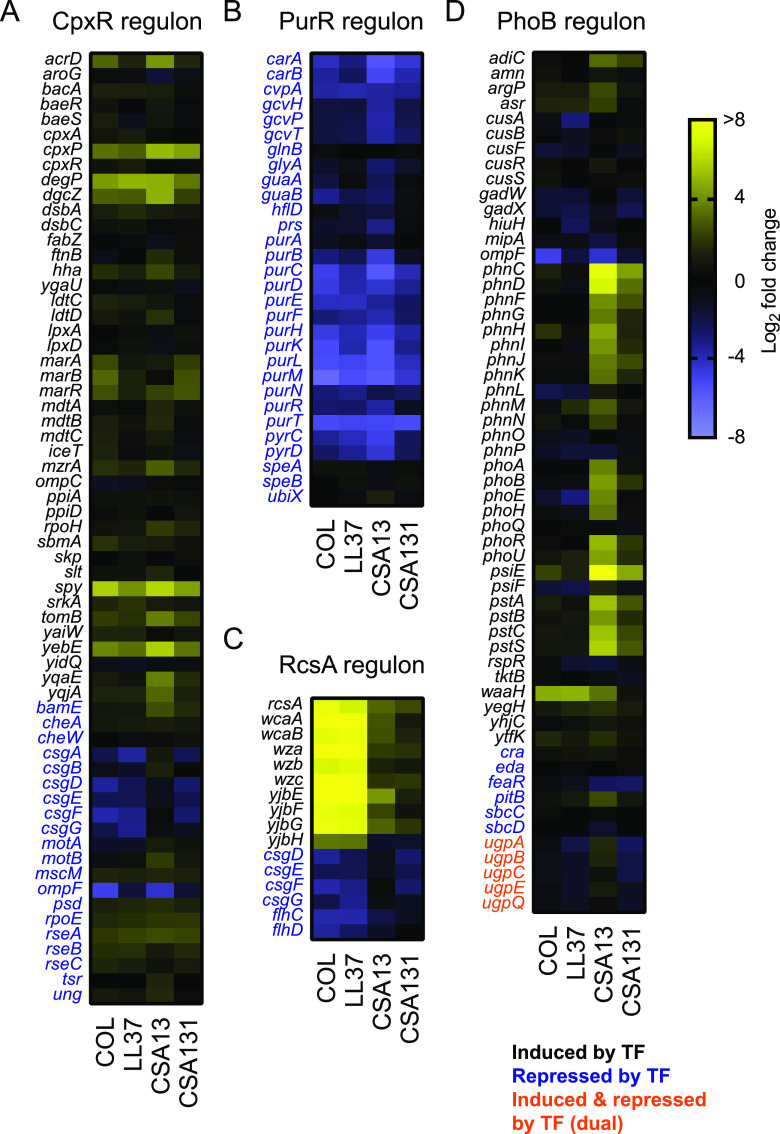
Expression of the CpxR, PurR, RcsA, and PhoB regulons in E. coli exposed to ceragenins. Heat maps of log_2_-fold change for genes of the CpxR (A), PurR (B), RcsA (C), and PhoB (D) regulons in bacteria exposed to antibiotics. Genes predicted to be induced (in black), repressed (in blue) or both (in orange) by specific transcription factors are indicated. Data are from 3 independent experiments.

To further confirm a role for CpxR and PurR in the response of E. coli to CAMPs and ceragenins, the expression of the CpxR and PurR regulons was analyzed in more detail (Fig. 5A and B and Data Set S4). The heat map of the CpxR regulon showed a consistent regulation of several genes in bacteria exposed to all four compounds (e.g., *cpxP*, *degP*, *dsbA*, *spy*, and *yebE*) (Fig. 5A) and corroborated our results described above (Fig. 3B and 4A). Likewise, most of the genes of the PurR regulon were also downregulated under these conditions (Fig. 5B), again consistent with our findings described above (Fig. 2K, 3C and D, and 4B). These results confirmed that the expression of the CpxR and PurR regulons are modulated in E. coli exposed to CAMPs and ceragenins.

Our expression analysis led us to focus on the RcsA and PhoB regulons to gain insight into the differential regulation of genes involved in the biosynthesis of colanic acid and phosphate transport, respectively (Fig. 5C and D and Data Set S4). RcsA regulates the expression of genes involved in colanic acid biosynthesis (21), a pathway that was upregulated in E. coli exposed to CAMPs, but not ceragenins (Fig. 2J, and 3A and B). Accordingly, the heat map of the RcsA regulon showed a marked modulation of this pathway in E. coli exposed to CAMPs in comparison to ceragenins (Fig. 5C). The phosphate regulon transcriptional regulatory protein PhoB regulates the expression of genes involved in phosphate transport (29) and was upregulated in E. coli following exposure to ceragenins, but not CAMPs (Fig. 2J, and 3A and B). Accordingly, the heat map of the PhoB regulon showed partial but specific induction in bacteria exposed to ceragenins (Fig. 5D). More specifically, upregulation of the *phnC-phnP* operon, the *pstSCAB-phoU* operon as well as a trend for other *pho* genes (e.g., *phoB* and *phoR*) and the phosphate starvation-inducible *psiEF* genes were specifically observed in bacteria exposed to ceragenins (Fig. 5D). These results suggested that the specific upregulation of genes involved in colanic acid biosynthesis and phosphate transport in bacteria exposed to CAMPs and ceragenins are mediated by RcsA and PhoB, respectively.

### Proteomic response of E. coli exposed to colistin and CSA13.

**FIG 6:**
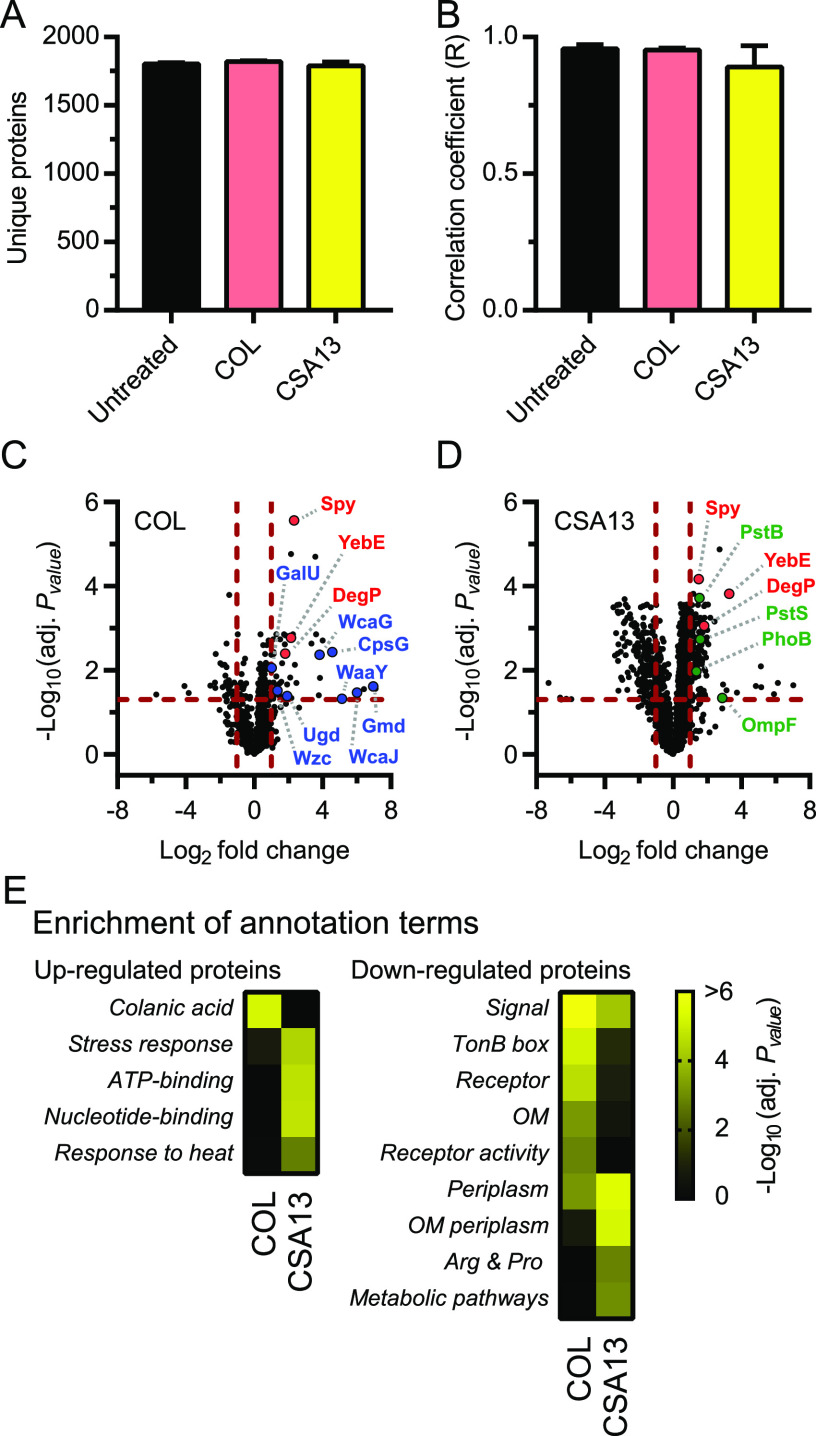
Proteomic response of E. coli exposed to colistin and CSA13. (A) The number of unique proteins identified for each condition. (B) Correlation coefficient (R) for the number of peptides per protein between the biological replicates of untreated bacteria and bacteria exposed to colistin (COL) or CSA13. Data are represented as means and standard deviations. (C and D) Volcano plots that represent protein expression as means of log_2_-fold change and −log_10_ adjusted *P* values (adj. *P* value) for bacteria exposed to antibiotics in comparison to untreated controls. Horizontal and vertical dotted red lines indicate adjusted *P* values less than 0.05 (or −log_10_ [adj. *P* value] greater than 1.3) and absolute log_2_-fold change greater than 1. Some upregulated proteins that are members of the Cpx regulon (in red), involved in colanic acid and LPS biosynthesis (in blue), or members of the PhoB regulon (in green) are highlighted. (E) Annotation terms enriched for proteins significantly up- or downregulated (absolute log_2_ FC >1 and adj. *P* value <0.05) following the exposure of E. coli to colistin and CSA13. Adjusted *P* values of annotation terms associated with a false discovery rate (FDR) value >0.05 for at least one antibiotic are shown. Annotation terms are abbreviated and/or modified for a purpose of presentation (see Data Set S6 for original annotation terms). Data are from 3 independent bacterial cultures for each condition.

To validate the previous conclusions as well as to determine if the changes in mRNA levels in response to the molecules led to changes in the proteome, we measured global protein abundance in bacteria exposed to colistin and CSA13 by mass spectrometry-based proteomics. E. coli cultures were grown to log phase and treated with supra-MICs of antibiotics before protein extraction, peptide preparation, and peptide quantification (see Materials and Methods). Approximately 1800 unique proteins were detected for each biological replicate (Fig. 6A) and the number of unique peptides identified showed an excellent correlation between biological replicates (Fig. 6B). Several statistically significant changes in protein expression (absolute log_2_-fold change >1 and adjusted *P* value <0.05) were observed following exposure of E. coli to colistin (Fig. 6C) and CSA13 (Fig. 6D) (see Data Set S5). This dataset showed that E. coli exposed to both colistin and CSA13 upregulated the proteins DegP, Spy, and YebE (Fig. 6C and D, and Table S2), which are known members of the Cpx regulon (19) that were strongly upregulated at the transcriptional level following exposure to CAMPs and ceragenins (Fig. 5A and Table S2). The dataset also revealed modulation of proteins involved in colanic acid and LPS biosynthesis (e.g., Ugd and WcaG) (Fig. 6C and Table S2) or related to the PhoB regulon (e.g., PstB and PstS) (Fig. 6D and Table S2) in bacteria exposed to colistin or CSA13, respectively, which also corroborate our transcriptional data (Fig. 5C and D, and Table S2). Therefore, the proteomic data are consistent with our transcriptional analysis and revealed a common induction of the Cpx envelope stress response in response to CAMPs and ceragenins. The results also corroborated that colistin and CSA13 specifically induced proteins involved in colanic acid biosynthesis and modulated members of the PhoB regulon, respectively.

10.1128/mBio.02726-21.2 TABLE S2 Comparison of the transcriptional and protein responses of E. coli exposed to colistin and CSA13 for selected genes. Changes in protein and RNA abundance (log_2_ FC) and adjusted *P* values (adj. *P* value) are shown. Genes were selected based on significant upregulation ( *P* value <0.05) at the protein level for the following categories: CpxR regulon proteins upregulated by colistin and CSA13, colanic acid or LPS biosynthesis proteins upregulated by colistin, PhoB regulon proteins upregulated by CSA13 and heat response proteins upregulated by CSA13. Download Table S2, PDF file, 0.3 MB. Copyright © 2022 Mitchell et al. 2022 Mitchell et al. https://creativecommons.org/licenses/by/4.0/ This content is distributed under the terms of the Creative Commons Attribution 4.0 International license.

10.1128/mBio.02726-21.8 DATA SET S5 Proteomic responses of E. coli exposed to colistin and CSA13. This file includes fold changes and adjusted *P* values (including imputed values) as well as lists of proteins significantly up- or down-regulated following exposure to colistin and CSA13. Download Data Set S5, XLSX file, 0.2 MB. Copyright © 2022 Mitchell et al. 2022 Mitchell et al. https://creativecommons.org/licenses/by/4.0/ This content is distributed under the terms of the Creative Commons Attribution 4.0 International license.

10.1128/mBio.02726-21.9 DATA SET S6 Annotation term enrichment analysis for proteins up- or down-regulated in E. coli exposed to colistin or CSA13. The analysis was performed with the DAVID Bioinformatics Resources using terms from UniProt keywords, COG ontology, GO, and the KEGG pathway. This dataset also includes Venn diagram lists and annotation term enrichment analysis for proteins significantly up- or down-regulated in E. coli by both colistin and CSA13, only colistin or only CSA13 (bioinformatics resources were accessed on 2021-11-04 for the latter analysis). Download Data Set S6, XLSX file, 0.1 MB. Copyright © 2022 Mitchell et al. 2022 Mitchell et al. https://creativecommons.org/licenses/by/4.0/ This content is distributed under the terms of the Creative Commons Attribution 4.0 International license.

Annotation term enrichment analysis was performed on the proteomic dataset to identify pathways significantly modulated in bacteria exposed to colistin and CSA13 (see Data Set S6). Similar to our transcriptional results (Fig. 2J), the colanic acid pathway was significantly enriched among proteins upregulated by colistin but not CSA13 (Fig. 6E). A heat response signature was significantly enriched among proteins upregulated by CSA13 but not colistin (Fig. 6E), corroborating the transcriptional upregulation of genes associated with this pathway and with cis-acting elements for σ^H^ (Fig. 2J and 4A, and Table S2). In addition, while the periplasm pathway was enriched among proteins downregulated by both colistin and CSA13, the outer membrane pathway was significantly enriched among proteins downregulated by colistin but not by CSA13 (Fig. 6E). Overall, these results confirmed that E. coli responded distinctly to colistin and CSA13, although both compounds modulated proteins associated with the bacterial envelope. Thus, while both molecules triggered a common set of genes involved in bacterial envelope stress, they also activated distinct response pathways, indicating they have overlapping but not identical modes of action.

### Identification of genetic determinants of resistance to ceragenins in E. coli.

Our inability to identify ceragenin-resistant E. coli mutants (Fig. 1L and M) was consistent with results previously published by Pollard et al. (13) and indicated that resistance to ceragenins emerged infrequently in culture despite strong selective pressure. This suggested that ceragenins either have multiple essential targets or affect cellular structures that are immutable. Thus, traditional genetic approaches to identify the target(s) of ceragenins have not been feasible. To gain insight into the genetic determinants of ceragenin action, we employed an alternative genetic approach that utilizes CRISPRi to reduce the expression of genes in E. coli. As demonstrated previously, this approach allows for partial knockdown of essential E. coli genes, practically creating hypomorphic alleles that have reduced function but still promote cell viability (30, 31). By combining these genetic perturbations with subinhibitory concentrations of antibiotics and by measuring the effects on bacterial fitness, we sought to identify functional interactions between bacterial pathways and antibiotic stress.

**FIG 7:**
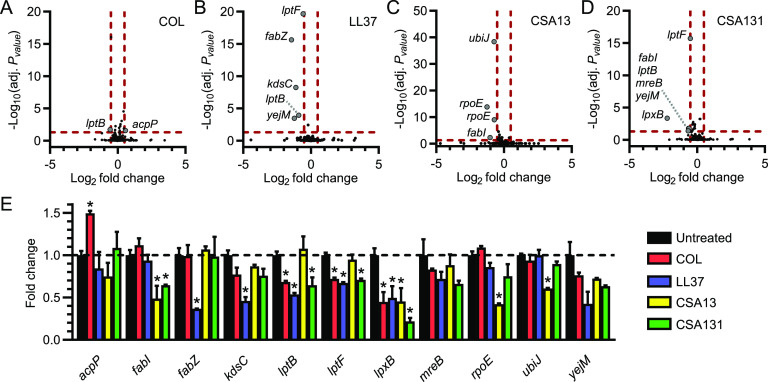
Identification of the genetic determinants of resistance to ceragenins in E. coli using CRISPRi. A pooled CRISPRi library that allows the inducible knockdown of predicted essential genes was used to study the genetic determinants of resistance to CAMPs and ceragenins. Changes in abundance (Log_2_FC) and adjusted *P* values (−log_10_ [adj. *P* value]) associated with each strain following exposure to colistin (COL) (A), LL37 (B), CSA13 (C), and CSA131 (D) are shown. Horizontal and vertical dotted red lines indicate adjusted *P* values less than 0.05 (or−log_10_ [adj. *P* value] greater than 1.3) and absolute log_2_-fold change greater than 0.5. Genes associated with significant changes in abundance are labeled for each compound. (E) Mean fold changes in abundance and standard deviations (SD) associated with significantly enriched or depleted CRISPRi strains (only one *rpoE*-targeting strain is shown) following exposure to COL, LL37, CSA13, and CSA131 (*, *P* <0.05 [two-tailed unpaired *t* test]). Means and SDs were calculated from counts normalized to the total number of counts for each condition. Data are from two biological replicates.

We screened a pooled library of inducible knockdown strains targeting essential genes (30) grown with or without subinhibitory concentrations of CAMPs or ceragenins. To evaluate the fitness of each CRISPRi knockdown within the complex population, we used deep sequencing to measure the relative abundance of guide sequences during the different culturing conditions. Log_2_-fold changes (log_2_ FC) in abundance in response to CAMPs and ceragenins were calculated in comparison to the control condition for each knockdown strain, and significantly resistant or sensitized strains (defined by absolute log_2_-fold change > 0.5 and an adjusted *P* value <0.05) were identified for each of the compounds (Fig. 7A to D and Data Set S7). Although no clear correlation was observed between hits identified using the CRISPRi approach and the transcriptional responses analyzed above (Table S3), CRISPRi strains depleted or enriched following exposure to one or several antimicrobials were predominantly knocked down for genes involved in the bacterial envelope. This provided further evidence that the bacterial envelope is the cellular structure targeted by both CAMPs and ceragenins.

10.1128/mBio.02726-21.3 TABLE S3 Transcriptional responses of E. coli exposed to colistin, LL37, CSA13, and CSA131 for the genetic determinants of antimicrobial resistance identified using CRISPRi. Transcriptional responses were determined using the RNAseq dataset. Download Table S3, PDF file, 0.08 MB. Copyright © 2022 Mitchell et al. 2022 Mitchell et al. https://creativecommons.org/licenses/by/4.0/ This content is distributed under the terms of the Creative Commons Attribution 4.0 International license.

10.1128/mBio.02726-21.10 DATA SET S7 Changes in abundance and statistical significance associated with each strain of the CRISPRi library following exposure to colistin, LL37, CSA13, and CSA131. Download Data Set S7, XLSX file, 0.1 MB. Copyright © 2022 Mitchell et al. 2022 Mitchell et al. https://creativecommons.org/licenses/by/4.0/ This content is distributed under the terms of the Creative Commons Attribution 4.0 International license.

The changes in abundance for the genes identified above were further analyzed and compared between treatments (Fig. 7E). Several genes involved in the LPS biosynthetic pathway were identified in these screens, and knockdown of the lipid-A-disaccharide synthase LpxB (32) sensitized E. coli to all the compounds, highlighting the bacterial surface as a common site of action for CAMPs and ceragenins. Silencing of the LPS transport genes *lptB* and *lptF* (33) led to sensitivity to colistin, LL37, and CSA131 but not to CSA13, which corroborates the above transcriptomic data and suggests distinctive mechanisms of action for CSA13 and CSA131 (Fig. 2I to K). Knockdown of *kdsC* (34), encoding an enzyme involved in LPS biosynthesis, only led to sensitivity to LL37. Knockdown strains for *rpoE*, the gene encoding the envelope stress-responsive sigma factor σ^E^ (21, 24, 25), and the ubiquinone biosynthesis gene *ubiJ* (35) were sensitive to CSA13 but not to CSA131, which also supports the idea that these ceragenins act distinctively on bacteria. On the other hand, the knockdown strain for the fatty acid biosynthesis gene *fabI* (36, 37) was sensitive to both CSA13 and CSA131 and not to the CAMPs and may constitute a common molecular determinant of sensitivity to ceragenins. Other genes involved in fatty acid metabolism were identified as genetic interactors with CAMPs. More specifically, interference with the expression of *acpP*, encoding the acyl carrier protein (38), led to resistance to colistin, whereas knockdown of *fabZ*, encoding a lipid dehydratase (39), led to sensitivity to LL37, indicating differences between the mechanisms of action of these two CAMPs, as previously suggested (16, 40). Taken together, these results support the notion that both CAMPs and ceragenins work by similar yet distinct mechanisms. These studies also provide a starting point for the genetic determination of the mode of action of ceragenins.

## DISCUSSION

Although ceragenins were designed to mimic the physiochemical properties of CAMPs (11, 12), our results indicated that they evoke different responses from bacteria than naturally occurring CAMPs. Our finding that ceragenins are active against a broader array of microbes than CAMPs, including mycobacteria, was our first indication that they have different mechanisms of action. Using transcriptomics, proteomics, and a CRISPRi genetic approach, we compared the responses of bacteria to CAMPs and ceragenins and revealed similarities, but also striking differences, and showed that ceragenins trigger a distinctive envelope stress response. Furthermore, our data also suggested that the two prototypical ceragenins, CSA13 and CSA131, trigger different responses in bacteria. Overall, while our results confirmed that ceragenins act on the bacterial envelope, they challenged the assumption that CAMPs and ceragenins share the same mechanism of action.

Profiling the responses of E. coli to CAMPs and ceragenins showed that these compounds trigger the Cpx envelope stress response, which is known to contribute to the bacterial adaptation to defects in the secretion and folding of the inner membrane and periplasmic proteins (21, 41). This corroborates previous studies demonstrating that the CpxR/CpxA system influences the susceptibility of bacteria to CAMPs (42, 43) and suggests that the Cpx response might similarly help bacteria survive exposure to ceragenins. The hypothesis that the envelope stress response is induced by CAMPs and ceragenins is also supported by the modulation of genes associated with cis-acting elements for σ^E^ (Fig. 4A) and by the enrichment and/or depletion of CRISPRi strains targeting components of the bacterial envelope (Fig. 7). However, additional mechanistic studies are required to establish if regulators of the envelope stress response (e.g., CpxR, CpxA, and σ^E^) influence the susceptibility of E. coli to ceragenins and directly contribute to the bacterial response.

A striking similarity between the transcriptomic profiles of bacteria exposed to CAMPs and ceragenins is the downregulation of genes involved in the biosynthesis of purines and pyrimidines (Fig. 2K, 3C and D, 4B, and 5B). The cause of this downregulation is unknown, but it is possible that the repression of these metabolic pathways is part of the adaptive response to antibiotic exposure (44) and/or relates to a decreased requirement for nucleic acid in growth-inhibited bacteria. An intriguing question is whether the flux of the metabolites through these nucleotide metabolic pathways affects susceptibility to antimicrobial agents targeting the bacterial envelope, as observed for other antibiotics (45).

The results of this study showed that E. coli responds differently to CAMPs and ceragenins. We showed that CAMPs specifically induced the Rcs response and the expression of genes involved in the biosynthesis of colanic acid (Fig. 2J, 3A and B, 5C, and 6C and E). This is consistent with a previous study that demonstrated that CAMPs, including polymyxin B and LL37, induce the Rcs regulon through the outer membrane lipoprotein RcsF (46). Surprisingly, the ceragenins CSA13 and CSA131 did not induce the Rcs response as markedly as CAMPs (Fig. 5C). Thus, in contrast with the current model that outer membrane perturbation by CAMPs is required for the activation of the Rcs response by RcsF (46, 47), our results show that ceragenins perturb the bacterial envelope of E. coli without extensively triggering the Rcs response. We also found that ceragenins, but not CAMPs, induced the expression of genes involved in phosphate transport and of the PhoB regulon (Fig. 2J, 3A and B, 5D, and 6D). Although the reasons why these genes are differently modulated following exposure to antimicrobial compounds are not understood and warrant additional mechanistic studies, these results strongly suggest that ceragenins and CAMPs have distinctive mechanisms of action.

Despite some similarities between the response of bacteria exposed to ceragenins, such as the upregulation of several genes of the Cpx and PhoB regulons and the downregulation of genes involved in nucleotide metabolism, our data also showed striking differences between bacteria exposed to CSA13 and CSA131 (Fig. 2I to K and 7E). These differences include the upregulation of genes encompassing several functions (e.g., transcription factors and proteins involved in the heat response) as well as the downregulation of genes involved in protein translation in bacteria exposed to CSA13 (Fig. 2J and K). While the basis for these differences is unknown, as noted above, the LogP values of CSA13 and CSA131 are almost 2 orders of magnitude apart, suggesting that the level of hydrophobicity of the compounds may underlie their different effects. Given that the site of action is the bacterial envelope, such a significant difference in partition coefficient values is likely to alter responses to membrane targets, especially the extremely hydrophobic outer membrane of mycobacteria. Interestingly, results from a previous study demonstrated that, unlike other ceragenins, CSA13 can permeabilize both the outer and inner membranes of E. coli (14). Although CSA131 was not included in this previous study, one hypothesis is that differences in membrane permeabilization by CSA13 and CSA131 have an impact on the bacterial response to these antimicrobials. Future work exploring the response of bacteria to a broader range of ceragenins will help in understanding these differences and might guide the design of compounds with a more specific mode of action.

Our CRISPRi approach identified sensitizing interactions between genes involved in the biology of the bacterial envelope and the antibacterial compounds colistin, LL37, CSA13, and CSA131 (Fig. 7). This information might prove valuable for the design of combination therapies that are synergistic and prevent the emergence of resistance but also allow treatment regimens with lower concentrations of antibiotics and dose-related antibiotic toxicity (48–50). As an example, triclosan, a compound that inhibits the ceragenin-sensitivity determinant FabI (51) (Fig. 7E), might synergize with ceragenins by altering the properties of the bacterial envelope and sensitizing bacteria to the action of ceragenins. As another example, the CAMPs/ceragenins-sensitivity determinant LpxB was suggested as a target for the development of antibacterial compounds (52), which compounds would have the potential to synergize with CAMPs and ceragenins more broadly (Fig. 7E), assuming that the target for CAMPs and ceragenins is not LpxB. Although those antibacterial interactions are purely speculative, our results suggest that CRISPRi approaches could be valuable tools for target identification and the development of antibiotic combination therapies.

The results of this study suggested that CAMPs and ceragenins both kill bacteria by targeting the bacterial envelope. However, this study also supports the hypothesis that ceragenins have a distinctive mode of action and we propose a model in which ceragenins cross the outer layers of the bacterial envelope and target more specifically the inner membrane. This hypothesis is supported by the broad spectrum of action of these molecules, which extend beyond bacteria. Whether the broader activity range of ceragenins impacts the selectivity for microbial membranes characteristic of endogenous CAMPs remains a key question for future study. A better understanding of the structure-activity relationship of these compounds and a deeper knowledge of their unique mechanism of action will be essential in the discovery of the next generation of ceragenins with increased potency and selectivity. Future studies should also focus on characterizing the response and the genetic determinants of resistance to ceragenins in mycobacteria and Gram-positive bacteria as the identification of the target(s) of ceragenins may lead to the development of broad-spectrum therapeutics against bacterial diseases.

## MATERIALS AND METHODS

### Antimicrobial compounds.

CSA13 and CSA131 (53) as well as CSA44 and CSA144 (54) were prepared as described previously and solubilized at 10 mg/mL in sterile distilled and deionized (DD) water. LL37 (Anaspec, Fremont, CA, USA), colistin (Sigma-Aldrich, St-Louis, MO, USA), and ciprofloxacin (MP Biomedicals, Irvine, CA, USA) were solubilized at 10 mg/mL in sterile DD water. Erythromycin (Sigma-Aldrich) was solubilized at 10 mg/mL in ethanol. Antimicrobial compounds were aliquoted and stored at −20°C. Freeze-thaw cycles of stock solutions were limited to three times.

### Bacterial strains and growth conditions.

E. coli MG1655 (55), L. monocytogenes 10403S (56), M. marinum strain M (57), M. smegmatis mc^2^155 (58), and M. tuberculosis Erdman (57) were as previously described. M. avium mc^2^2500 is a clinical strain isolated from an AIDS patient with pulmonary disease and predominantly formed a smooth/transparent colony morphotype on solid agar (15). M. avium mc^2^2500D is an isogenic, laboratory-derived strain with an opaque colony morphotype. E. coli and L. monocytogenes were routinely grown on Mueller-Hinton agar (MHA) (BD, Franklin Lakes, NJ, USA) plates or in cation-adjusted Mueller-Hinton (CAMH) (BD) broth and on Brain-heart infusion agar (BHIA) (Becton, Dickinson) plates or in Brain-heart infusion (BHI) (BD) broth, respectively. M. avium and M. tuberculosis were cultured in Middlebrook 7H9 (BD) broth containing 0.5% glycerol, 10% Oleic Albumin Dextrose Catalase (OADC) (Sigma-Aldrich), and 0.05% Tween 80. M. marinum was cultured in Middlebrook 7H9 broth containing 0.5% glycerol, 10% OADC, and 0.2% Tween 80. M. smegmatis was grown on Middlebrook 7H10 (BD) plates or Middlebrook 7H9 broth containing 0.5% glycerol, 0.5% dextrose, and 0.2% Tween 80 unless otherwise stated. All bacterial strains were grown at 37°C, except M. marinum, which was grown at 30°C. Liquid cultures were incubated with shaking unless otherwise stated.

### Antibiotic susceptibility testing.

MICs of antimicrobial compounds against E. coli and L. monocytogenes were determined by a broth microdilution technique following the recommendations of the Clinical and Laboratory Standards Institute (CLSI) (59), except that BHI was used to perform assays on L. monocytogenes. Antibiotic quality control experiments were performed using E. coli ATCC25922 (ATCC, Manassas, VA, USA). A similar protocol using extended incubation periods was used to determine MICs against M. avium (10 days), M. marinum (5 days), M. smegmatis (3 days), and M. tuberculosis (14 days). For the determination of MICs using mycobacterial species, plates were placed in a vented container containing damp wipes to minimize evaporation.

### Time-kill experiments.

Time-kill experiments were performed to characterize the effect of compounds on bacterial growth and survival. Bacteria were inoculated at 10^5^ to 10^6^ colony forming units (CFU)/mL in liquid media in the absence or presence of antibiotics at the following concentrations: E. coli, 0.5 μg/mL colistin, 64 μg/mL LL37, 4 μg/mL CSA13 and 4 μg/mL CSA131; L. monocytogenes, 2 μg/mL CSA13, 2 μg/mL CSA131, 2 μg/mL ciprofloxacin and 0.25 μg/mL erythromycin; M. smegmatis, 0.5 μg/mL CSA13 and 0.5 μg/mL ciprofloxacin. Bacterial cultures were grown at 37°C with shaking and the number of CFU/mL was determined at several time points. Plates without Tween 80 were used for the CFU determination of M. smegmatis cultures.

### Serial passage experiments.

Serial passage of bacteria in the presence of subinhibitory concentrations was performed as previously described (13, 60). Experiments were performed in CAMH or BHI broth for E. coli and L. monocytogenes, respectively. In a few cases, bacteria growing at the two highest subinhibitory concentrations of antimicrobial had to be combined to get inoculums of 10^5^ to 10^6^ CFU/mL.

### Preparation and sampling of bacterial cultures for transcriptomic profiling.

A single colony of E. coli MG1655 was inoculated into CAMH broth and incubated for 16 to 18 h at 37°C with shaking. Cultures were diluted in fresh media to an absorbance at 600 nm (A_600_) of 0.1, incubated at 37°C with shaking until an A_600_ of 0.8 to 1.0 (∼2 h) and antibiotics were added to each culture, which was further incubated for 1 h at 37°C with shaking. Antibiotics were adjusted to concentrations having a similar impact on E. coli growth for that particular, higher bacterial density, culture format (i.e., 4 μg/mL colistin, 8 μg/mL CSA13, 8 μg/mL CSA131, and 256 μg/mL LL37). Cultures samples were then mixed 1:2 with RNA Protect Bacteria Reagent (Qiagen, Germantown, MD, USA), vortexed immediately for 5 s, and incubated for 5 min at room temperature. The bacterial suspensions were centrifuged for 10 min at 5,000 × *g*, supernatants were discarded, and pellets were stored a few days at −80°C before proceeding to RNA extraction.

### RNA purification and sequencing.

Bacterial pellets were resuspended in 100 μL of 10 mM Tris, 1 mM EDTA, pH 8.0 buffer containing 10 mg/mL lysozyme (Sigma-Aldrich). Proteinase K (New England BioLabs, Ipswich, MA, USA) (2.5 μL of 20 mg/mL) was added and samples were incubated at room temperature for 10 min with frequent mixing. Samples were combined with 0.5 μL of 10% SDS and 350 μL of lysis buffer (Ambion life technologies, Invitrogen, Carlsbad, CA, USA) containing β-mercaptoethanol, vortexed and lysates were transferred into 1.5 mL RNase-free microcentrifuge tubes. Samples were then passed 5 times through an 18 to 21-gauge needle and centrifuged at 12,000 × *g* for 2 min at room temperature. Supernatants were transferred to new 1.5 mL RNase-free microcentrifuge tubes before proceeding to the washing and elution steps described in the PureLink RNA minikit (Ambion Life Technologies). Samples were treated with DNase (New England BioLabs) for 15 min at 37°C in a volume of 50 μL and 5 μL of 25 mM EDTA was added. Samples were further incubated for 10 min at 75°C and quickly placed on ice before being cleaned and reeluted using the RNA Clean & Concentrator kit (Zymo Research, Irvine, CA, USA) and stored at −80°C. The quality and the quantity of each RNA sample were analyzed by the UC Berkeley QB3 facility using a bioanalyzer and the Qubit technology. RNA samples were sequenced and preliminarily analyzed by the University of California (UC) Davis Genome Center and the UC Davis Bioinformatics Core.

### Analysis of RNAseq data.

The differential expression analyses were conducted using the limma-voom Bioconductor pipeline (61) (EdgeR version 3.20.9, limma version 3.34.9) and R 3.4.4 by the UC Davis Bioinformatics Core. The multidimensional plot was created using the EdgeR function plotMDS. Pathway analyses were performed using DAVID Bioinformatics Resources 6.8 (62, 63). Only annotation terms from the following databases were included: UP (UniProt) Keywords, COG (cluster of orthologous groups) Ontology, GO (gene ontology for biological process, molecular function, and cellular component), and KEGG. Venn diagram analyses were performed using the tool provided on the Bioinformatics & Evolutionary Genomics website of Ghent University (64). Promoter and regulatory binding analyses were performed using the Gene Expression Analysis Tools (20). Only one repeated binding site and promoter was considered for each gene for any specific transcription factors to avoid redundancy. Lists of genes to be included in specific regulons were retrieved from the RegulonDB Database (65). Fold changes for few transcripts of regulons CpxR (*cpxQ*, *csgC*, *cyaR*, *efeU*, *rprA*, *rseD*), PurR (*codA*, *codB*) and PhoB (*cusC*, *phnE*, *prpR*) were not included in this analysis. The information related to the expected activity (induction and/or repression) of each transcription factor on specific genes was also retrieved from the RegulonDB Database.

### Protein extraction and peptide preparation from E. coli cultures.

A single colony of E. coli MG1655 was inoculated into CAMH broth and incubated for 16 to 18 h at 37°C with shaking. Cultures were diluted in fresh media to an A_600_ of 0.1, incubated at 37°C with shaking until an A_600_ of 0.8 to 1.0 (∼2 h) and antibiotics (4 μg/mL colistin or 8 μg/mL CSA13) were then added to each culture, which was further incubated for 3 h at 37°C with shaking. Protein was extracted, digested, and desalted, as previously described (66), with few modifications. Briefly, 23 mL of the bacterial cultures were washed twice in cold PBS and resuspended in 4 mL of lysis buffer (8 M urea, 150 mM NaCl, 100 mM ammonium bicarbonate, pH 8) containing Roche mini-complete protease inhibitor EDTA-free and Roche PhosSTOP (1 tablet of each per 10 mL of buffer) (Roche, Basel, Switzerland). Samples (on ice) were then sonicated 10 times with a Sonics VibraCell probe tip sonicator at 7 W for 10 s. Insoluble precipitates were removed from lysates using a 30 min centrifugation at ∼16,100 × *g* at 4°C and the protein concentration of each lysate was determined using the microplate procedure of the Micro BCA^TM^ Protein assay kit (Thermo Fischer Scientific, Emeryville, CA, USA). Clarified lysates (1 mg each) were reduced with 4 mM tris(2-carboxyethyl)phosphine for 30 min at room temperature, alkylated with 10 mM iodoacetamide for 30 min at room temperature in the dark, and quenched with 10 mM 1,4-dithiothreitol for 30 min at room temperature in the dark. Samples were diluted with three volumes of 100 mM ammonium bicarbonate, pH 8.0, and incubated with 10 μg of sequencing grade modified trypsin (Promega, Madison, WI, USA) while rotating at room temperature for 18 h. Trifluoroacetic acid (TCA) was then added to a final concentration of 0.3% to each sample, followed by 1:100 of 6 M HCl and the removal of insoluble material by centrifugation at ∼2,000 × *g* for 10 min. SepPak C18 solid-phase extraction cartridges (Waters, Milford, MA, USA) were activated with 1 mL of 80% acetonitrile (ACN), 0.1% TFA, and equilibrated with 3 mL of 0.1% TFA. Peptides were desalted by applying samples to equilibrated columns, followed by a washing step with 3 mL of 0.1% TFA and elution with 1.1 mL of 40% ACN, 0.1% TFA. The subsequent global protein analysis was performed using 10 μg of each desalted peptide sample.

### Liquid chromatography, mass spectroscopy, label-free quantification, and analysis of the proteomic data.

Peptides were analyzed using liquid chromatography and mass spectroscopy, as previously described (66). Mass spectrometry data were assigned to E. coli sequences and MS1 intensities were extracted with MaxQuant (version 1.6.0.16) (67). Data were searched against the E. coli (strain K-12) protein database (downloaded on November 6, 2018). MaxQuant settings were left at the default except for that trypsin (KR|P) was selected, allowing for up to two missed cleavages. Data were then further analyzed with the artMS Bioconductor package (68), using the MSstats Bioconductor package (version 3.14.1) (69) and the artMS version 0.9. Contaminants and decoy hits were removed, and samples were normalized across fractions by median-centering the log_2_-transformed MS1 intensity distributions. The MSstats group comparison function was run with no interaction terms for missing values, no interference, unequal intensity feature variance as well as the restricted technical and biological scope of replication. Log_2_-fold change for proteins/sites with missing values in one condition but found in >2 biological replicates of the other condition of any given comparison were estimated by imputing intensity values from the lowest observed MS1-intensity across samples (68), and *P* values were randomly assigned between 0.05 and 0.01 for illustration purposes. Pathway and Venn diagram analyses were performed as described for the analysis of RNAseq data.

### Identification of genetic determinants of resistance to antibiotics using clustered regularly interspaced short palindromic repeats interference (CRISPRi).

A pooled CRISPRi library of ∼500 strains that allow the inducible knockdown of genes predicted to be essential was used to study the genetic determinants of resistance to CAMPs and ceragenins, as previously described (30), with few modifications. To quantify the antibiotic sensitivity of each CRISPRi strain, the relative proportion of each sgRNA spacer in the mixed population was enumerated by deep sequencing, after 15 doublings in the presence of saturating IPTG and 0.031 μg/mL colistin, 12 μg/mL LL37, 0.5 μg/mL CSA13 or 0.25 μg/mL CSA131. Experiments were run in parallel for two studies, and a detailed description of Materials and Methods as well as data for the untreated controls with or without IPTG-induction was described previously (30).

### Determination of log(P) values.

Log P values (partition coefficient) were determined using Chemicalize from ChemAxon (Escondido, CA, USA).

### Preparation of graphs.

GraphPad Prism software (v.7.00) was used to generate graphs and perform statistical tests. The number of independent experiments is indicated in each figure legend.

### Data availability.

RNAseq data were deposited in the GEO repository under the GEO accession number GSE160082. DNA sequencing data obtained with the pooled CRISPRi library were deposited in the Short Read Archive under accession number PRJNA669343. The mass spectroscopy proteomics data were deposited to the ProteomeXchange Consortium via the PRIDE (70) partner repository with the dataset identifier PXD022149.
