# Supplementary material for: Ceragenins and Antimicrobial Peptides Kill Bacteria through Distinct Mechanisms
Source: mBio. 2022 Jan 25;13(1):e02726-21. doi: 10.1128/mbio.02726-21 (PMC8787472; doi:10.1128/mbio.02726-21)
Supplement: TABLE S1 [file mbio.02726-21-st001.pdf]

TABLE S1. Susceptibility of *E. coli*, *L. monocytogenes* and *Mycobacterium* spp. to antibiotics

Specie	Strain	MIC[a] (μg/mL)						
		COL	LL37	CSA13	CSA131	CSA44	CSA144	CIP
E. coli	MG1655	0.25-1	16-32	2-4	2-4	2-4	4-16	0.015
L. monocytogenes	10403S	128- >128	32-64	1-2	1-2	2-4	4-8	1
M. avium	mc^{2}2500	>128	>128	128- >128	>128	ND	ND	0.5-2
	mc^{2}2500D6	>128	>128	32	64	ND	ND	0.125-0.25
M. marinum	M	>128	>128	32-128	64-128	ND	ND	0.25-1
M. smegmatis	mc^{2}155	64-128	>128	0.125-0.25	0.5	16-32	64-128	0.25
M. tuberculosis	Erdman	128- >128	>128	16	64	ND	ND	0.5

[a] COL, colistin; CIP, ciprofloxacin; ND, not determined.
