# Supplementary material for: Ceragenins and Antimicrobial Peptides Kill Bacteria through Distinct Mechanisms
Source: mBio. 2022 Jan 25;13(1):e02726-21. doi: 10.1128/mbio.02726-21 (PMC8787472; doi:10.1128/mbio.02726-21)
Supplement: TABLE S2 [file mbio.02726-21-st002.pdf]

TABLE S2. Comparison of the transcriptional and protein responses of *E. coli* exposed to colistin and CSA13 for selected genes[a]

Gene symbol	Protein				RNA			
	Colistin		CSA13		Colistin		CSA13	
	Log_2FC	Adj. P_{value}	Log_2FC	Adj. P_{value}	Log_2FC	Adj. P_{value}	Log_2FC	Adj. P_{value}
CpxR regulon[b]								
degP	1.81	<0.05	1.81	<0.05	4.36	<0.05	4.81	<0.05
spy	2.34	<0.05	1.50	<0.05	5.67	<0.05	5.96	<0.05
yebE	2.17	<0.05	3.29	<0.05	3.89	<0.05	5.70	<0.05
Colanic acid and LPS[c]								
cpsG	4.57	<0.05	0.96	NS	6.96	<0.05	0.91	NS
galU	1.03	<0.05	-0.13	NS	1.71	<0.05	-1.21	<0.05
gmd	6.96	<0.05	ND	ND	8.84	<0.05	2.35	<0.05
ugd	1.93	<0.05	0.06	NS	4.36	<0.05	-1.03	NS
waaY	5.15	<0.05	5.91	<0.05	0.32	NS	-0.80	NS
wcaG	3.84	<0.05	ND	ND	9.00	<0.05	1.76	NS
wcaJ	6.02	<0.05	ND	ND	7.44	<0.05	1.74	NS
wzc	1.36	<0.05	-0.44	NS	8.52	<0.05	1.84	NS
PhoB regulon[d]								
ompF	1.19	NS	2.88	<0.05	-4.93	<0.05	-4.33	<0.05
phoB	-0.16	NS	1.37	<0.05	-0.45	NS	4.31	<0.05
pstB	0.71	<0.05	1.55	<0.05	0.60	NS	4.21	<0.05
pstS	-0.62	NS	1.60	<0.05	0.71	NS	5.77	<0.05
Heat response[e]								
clpA	0.04	NS	1.03	<0.05	1.12	<0.05	2.90	<0.05
clpB	0.02	NS	1.30	<0.05	0.75	NS	4.57	<0.05
degP	1.81	<0.05	1.81	<0.05	4.36	<0.05	4.81	<0.05
hslU	0.31	NS	1.00	<0.05	0.39	NS	1.26	<0.05
hslV	0.08	NS	1.68	<0.05	0.33	NS	1.91	<0.05
htpG	0.28	NS	1.21	<0.05	0.30	NS	2.00	<0.05
ibpA	0.17	NS	3.14	<0.05	-0.07	NS	5.02	<0.05
ibpB	-0.09	NS	5.15	<0.05	-0.22	NS	7.23	<0.05
lon	0.31	NS	1.05	<0.05	0.28	NS	1.26	<0.05
nudF	0.56	NS	1.12	<0.05	-0.22	NS	-0.40	NS
pspA	1.79	<0.05	1.81	<0.05	2.11	<0.05	5.36	<0.05
rhlE	0.30	NS	1.84	<0.05	0.88	NS	-0.58	NS

[a] Log_2FC, Log_2 fold change; Adj. P_{value}, adjusted P_{value}; ND, not determined; NS, not significant.

[b] CpxR regulon genes significantly upregulated at the protein level in *E. coli* exposed to colistin and CSA13.

[c] Genes involved in colanic acid (GO:0009242) and lipopolysaccharide (LPS) (GO:0009103) biosynthetic processes and significantly upregulated at the protein level in *E. coli* exposed to colistin.

[d] PhoB regulon genes significantly upregulated at the protein level in *E. coli* exposed to CSA13.

[e] Genes involved in the heat response (GO:0009408) and significantly upregulated at the protein level in *E. coli* exposed to CSA13.
