# Supplementary material for: Ceragenins and Antimicrobial Peptides Kill Bacteria through Distinct Mechanisms
Source: mBio. 2022 Jan 25;13(1):e02726-21. doi: 10.1128/mbio.02726-21 (PMC8787472; doi:10.1128/mbio.02726-21)
Supplement: TABLE S3 [file mbio.02726-21-st003.pdf]

TABLE S3. Transcriptional responses of *E. coli* exposed to antibiotics for the genetic determinants of resistance identified using CRISPRi[a]

Gene symbol	Colistin		LL37		CSA13		CSA131	
	Log_2FC	Adj. P_{value}	Log_2FC	Adj. P_{value}	Log_2FC	Adj. P_{value}	Log_2FC	Adj. P_{value}
acpP	-0.41	<0.05	-0.31	NS	0.14	NS	0.15	NS
fabI	0.13	NS	-0.11	NS	-0.93	<0.05	0.17	NS
fabZ	-0.13	NS	0.11	NS	-1.03	<0.05	0.35	NS
kdsC	0.22	NS	0.05	NS	0.11	NS	0.06	NS
lptB	0.46	NS	0.63	<0.05	-0.26	NS	0.26	NS
lptF	0.54	<0.05	0.48	<0.05	0.22	NS	0.34	NS
lpxB	-0.35	NS	-0.30	NS	-2.69	<0.05	-0.04	NS
mreB	0.09	NS	0.36	NS	-0.44	NS	0.54	NS
rpoE	1.53	<0.05	1.68	<0.05	1.88	<0.05	1.92	<0.05
ubiJ	-0.17	NS	0.66	<0.05	0.19	NS	0.06	NS
yejM	-0.05	NS	-0.25	NS	-0.18	NS	0.43	NS

[a] Log_2FC, Log_2 fold change; Adj. P_{value}, adjusted P_{value}; NS, not significant.
